# Tenofovir Hampers the Efficacy of Sorafenib in Prolonging Overall Survival in Hepatocellular Carcinoma

**DOI:** 10.3390/biomedicines9111539

**Published:** 2021-10-26

**Authors:** Kung-Hao Liang, Sung-Fang Chen, Yu-Hua Lin, Yu-De Chu, Yang-Hsiang Lin, Ming-Wei Lai, Chih-Lang Lin, Chau-Ting Yeh

**Affiliations:** 1 Department of Medical Research, Taipei Veterans General Hospital, Taipei 112, Taiwan; 2 Institute of Food Safety and Health Risk Assessment, National Yang Ming Chiao Tung University, Taipei 112, Taiwan; 3 Institute of Biomedical Informatics, National Yang Ming Chiao Tung University, Taipei 112, Taiwan; 4 Department of Chemistry, National Taiwan Normal University, Taipei 106, Taiwan; sfchen@ntnu.edu.tw (S.-F.C.); pink3356@hotmail.com (Y.-H.L.); 5 Liver Research Center, Chang Gung Memorial Hospital, Linkou, Taoyuan 333, Taiwan; yudechu19871003@gmail.com (Y.-D.C.); yhlin0621@cgmh.org.tw (Y.-H.L.); mingweilai@gmail.com (M.-W.L.); wn49792000@yahoo.com.tw (C.-L.L.); 6 Liver Research Unit, Keelung Chang Gung Memorial Hospital, Keelung 204, Taiwan; 7 Community Medicine Research Center, Keelung Chang Gung Memorial Hospital, Keelung 204, Taiwan; 8 Molecular Medicine Research Center, Chang Gung University, Taoyuan 333, Taiwan

**Keywords:** drug-drug interactions, glycoproteome, targeted therapy, entecavir, hepatitis B

## Abstract

Sorafenib is a first-line treatment for patients with advanced hepatocellular carcinoma (HCC). These patients may simultaneously receive anti-hepatitis B treatment if they are viremic. The *N-Acetylgalactosaminyltransferase 14* (*GALNT14*) gene can serve as a biomarker to guide HCC treatments. However, the enzyme substrates of its gene product, GalNAc-T14 (a glycosyltransferase), remained uncharacterized. Here, we conducted a glycoproteome-wide search for GalNAc-T14 substrates using lectin affinity chromatography followed by tandem mass spectrometry. Seventeen novel GalNAc-T14 substrates were identified. A connective map analysis showed that an antiviral drug, tenofovir, was the leading medicinal compound to down-regulate the expression of these substrates. In vitro assays showed that HCC cells were resistant to sorafenib if pretreated by tenofovir but not entecavir. Clinical analysis showed that the concomitant use of tenofovir and sorafenib was a previously unrecognized predictive factor for unfavorable overall survival (hazard ratio = 2.060, 95% confidence interval = [1.256, 3.381], *p* = 0.004) in a cohort of 181 hepatitis-B-related, sorafenib-treated HCC patients (concomitant tenofovir versus entecavir treatment; *p* = 0.003). In conclusion, by conducting a glycoproteome-wide search for GalNAc-T14 substrates, we unexpectedly found that tenofovir was a major negative regulator of GalNAc-T14 substrates and an unfavorable anti-hepatitis B drug in HCC patients receiving sorafenib.

## 1. Introduction

Chronic hepatitis B is a major etiology of hepatocellular carcinoma (HCC) worldwide [1,2,3,4,5,6,7,8,9,10,11] and has been responsible for ~80% of HCC in Taiwan before 1990 [12]. Anti-hepatitis B virus (HBV) treatments are routinely prescribed to viremic patients manifesting hepatitis flares [3,13,14,15]. Entecavir and tenofovir are the first-line oral antiviral drugs, which harbor high resistance barriers and can potently suppress viral replication to reduce liver necroinflammation [16,17,18,19,20]. On the other hand, the multikinase inhibitor sorafenib can prolong the overall survival in advanced stage HCCs [21,22]. It remained the only approved targeted therapy for late-stage HCC between 2007–2017 [23]. At this time, the drug is still being widely used worldwide, although lenvatinib and atezolizumab/bevacizumab have gradually taken over as the first-line treatments. Sorafenib and oral-antiviral drugs are often given simultaneously to HCC patients who have been HBV viremic. Most patients continue to receive oral antiviral drugs after the viral loads have been suppressed to undetectable levels by treatments, to prevent future viral reactivation due to the stopping of treatment [20]. Despite the concomitant use of sorafenib and oral-antiviral drugs, few investigations have been conducted on their mutual interfering effects.

We previously identified that genomic variants were associated with chemotherapeutic response in late-stage HCCs [24]. A series of studies showed that the genotype of a genomic variant rs9679162, located on the Polypeptide *N-Acetylgalactosaminyltransferase 14* (*GALNT14*) gene, was consistently associated with the treatment outcome of intermediate and advanced HCC [24,25,26,27,28,29]. Additionally, *GALNT14* genotypes were associated with the expression levels of their protein product, GalNac-T14, which had been shown to mediate the oncogenesis and/or treatment responses in several other cancers, including cholangiocarcinoma [30], colon cancer [31], esophageal cancer [32], neuroblastoma [33], lung cancer [34], and breast cancer [35]. These findings implicated the involvement of GalNac-T14 in cancer biology, offering effective biomarkers to guide anticancer treatments, such as choosing between systemic chemotherapy, hepatic arterial infusion chemotherapy, and sorafenib in HCCs [29]. *GALNT14* also possesses other functions less relevant to cancer. A genome-wide investigation of consanguineous families showed that damaging Mendelian mutations in *GALNT14* causes embryonic lethality, suggesting an irreplaceable role of GalNac-T14 in human development [36]. Germline mutations were also found in a recent study of the congenital disorders of glycosylation [37]. Finally, a genomic screening of familial neuroblastoma also identified function-disrupting germline mutations, which were responsible for the cancer occurrence [33].

*GALNT14* is the gene encoding for the GalNAc-T14 glycosyltransferase. Its major function is to enable post-translational glycosylation by adding N-acetyl-D- galactosamine residues to serine or threonine residues of its substrates [38]. The glycosylation then affected the cellular and physiological functions, which may underlie patients’ clinical outcome. Unfortunately, the enzyme substrates of GalNAc-T14, performing anticancer-related functions, remained largely unknown. Thus, we were motivated to conduct a glycoproteome-wide exploration of GalNAc-T14 substrates to further elucidate their clinical implications.

## 2. Materials and Methods

### 2.1. Establishing HCC Cell Lines with GalNAc-T14 Overexpression

The GALNT14 open reading frame DNA, amplified from cDNA of J7 cells using primers, forward: atcgGCGGCCGCatgcggcgcctgactcgtcg and reverse: atcgGCGGCCGCttaagagctcaccatgtccc, franking with the NotI cutting site (capitalized sequences), was inserted into pRC/CMV plasmid (Thermo Fisher Scientific, Waltham, MA, USA) using the restriction enzyme digestion/ligation method, which was then transfected to HCC cell lines, Huh7, J7, and Mahlavu, to generate stable GalNAc-T14 overexpressing cells. Corresponding mock control cell lines were also established with only the plasmid vector transfected. Western blotting using the antibody ab86526 (Abcam, Cambridge, UK) was performed to confirm the successful expression of GalNAc-T14 protein in the cells.

### 2.2. Identification of GalNAc-T14 Substrates

We employed the lectin-enriched proteomic approach to identify the GalNAc-T14 substrates expressed in the GalNAC-T14 overexpressing Huh7, J7, and Mahlavu cells but not (or less) in controls. Total proteins were extracted from these cells using the M-PER Mammalian Protein Extraction Reagent (Thermo Fisher Scientific, Waltham, MA, USA), in conjunction with HaltTM Protease Inhibitor Cocktail (Thermo Fisher Scientific, Waltham, MA, USA). Glycoproteins were then captured using a self-made lectin affinity GVS Centrex Centrifuge Filter (GVS Gruppo, Bologna, Italy), packed with Peanut Agglutin (PNA) and Vicia Villosa Lectin (VVA) (Vector Laboratories, Burlingame, CA, USA), which can bind to the carbohydrate sequence Gal-β(1-3)-GalNAc (N-acetylgalactosamine galactose, a.k.a. Thomsen–Friedenreich antigen, 365 daltons) and GalNAc (a.k.a. Tn antigen) respectively. The captured proteins were then eluted, subjected to dialysis, quantitated, trypsin-digested, desalted, and then identified using ultrahigh performance liquid chromatography/tandem mass spectrometry (UPLC-MS/MS).

UPLC was performed on NanoACQUITY (Waters, Milford, MA, USA) using a lab-made pre-column (100 μm × 2 cm) packed with C18 AQ 200 Å 5 mg (Michrom bioresources, CA, USA) dissolved in 1 mL methanol, followed by an analytical column (75 μm × 10 cm) packed with C18 100 Å, 5 mg (Macherey-Nagel, Düren, Germany) dissolved in 1 mL methanol. The mobile phase A was 0.1% formic acid (FA) in 100% H_2_O, while the mobile phase B was 0.1% FA in 100% acetonitrile. Samples were loaded to UPLC-MS/MS with a 140 min gradient of 2% B for 5 min, 2–40% B for 95 min, 40–80% B for 5 min, 80% B for 5 min, and 80–2% B for 5 min, followed by 25 min at 2% B.

**Figure 1:**
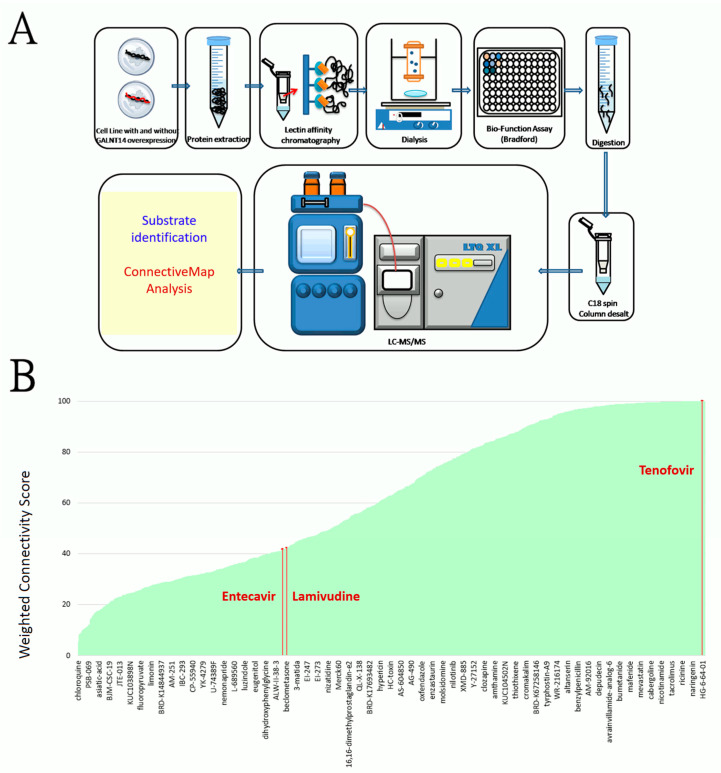
(**A**) The schematic diagram of the technical approach of detecting GalNAc-T14 substrates, comprising a lectin-affinity chromatography and UPLC-tandem mass spectrometry, followed by a protein identification procedure and a connective map analysis. (**B**) An extensive list of 1085 medicinal compounds (in the X-axis) ranked by their effects of down-regulating the GalNAc-T14 substrates. The effects were shown as the weighted connectivity score (marked in the Y axis). One representative compound per every 18 compounds were marked in the X axis to avoid an over complicated plot. The oral anti-HBV drug tenofovir is a leading compound for the suppression. Additionally, lamivudine ranked 723, while entecavir ranked 729 in the compound list sorted by the score.

Tandem mass spectrometry was performed on LTQ-XLTM (Thermo Fisher Scientific, Waltham, MA, USA), which was controlled by the Xcalibur (version 2.1) software. A 2.0 kV spray voltage was applied through a liquid junction. The temperature of the ion transfer capillary was set to 200 °C. A full scan was performed for ions in the range of m/z 350–2000. Precursor ions with multiple (2~4) charges then proceeded collision-induced dissociation to generate fragment ions for the identification. MSConvert was used to convert the mass spectrum into mgf files, then analyzed by the Mascot software with precursor mass tolerances of ±1.5 Da and fragment mass tolerances of ±0.6 Da (Figure 1A).

### 2.3. O-Glycosylation Site Prediction

We analyzed the O-GalNAc glycosylation sites of the identified GalNAc-T14 substrates using the NetOGlyc (4.0) online server at the site (https://services.healthtech.dtu.dk/service.php?NetOGlyc-4.0 (accessed on 20 June 2017)). This is a neural network based system that has learned the sequence features of the O-GalNAc glycosylation site detected by the Simple Cell technology [38]. The full-length protein sequences of the identified substrates were submitted to the online system, and the top hits were documented.

### 2.4. Connective Map Analysis

We used the online resource Connective Map at the following website (https://clue.io/cmap (accessed on 5 September 2019)) [39,40,41]. We entered the list of GalNAc-T14 substrates in the “Down-regulated genes” input box, asking the system to search for compounds that can suppress these genes. 

### 2.5. Patients

This study was approved by the institutional review board of Chang Gung Memorial Hospital, Taiwan, and conducted according to the ethical principles in the declaration of Helsinki. All patients were adults and had given informed consent. Clinical data from 181 patients (Jan 2010 to Jan 2020) were retrieved from the Liver Research Center for analysis. All these HCC patients were in Barcelona Clinic Liver Cancer (BCLC) stage C or in BCLC stage B but were not suitable for trans-arterial chemoembolization treatment. All patients received sorafenib treatment and were positive for HBV surface antigen. 

## 3. Results

### 3.1. Identification of Substrates of the GalNAc-T14 Glycosyltransferase

Substrates of the GalNAc-T14 glycosyltransferase were identified using a series of methods comprising lectin affinity chromatography, ultra-performance liquid chromatography, and tandem mass spectrometry (Figure 1A). We employed HCC cell lines (Huh7, J7 and Mahlavu) transfected by the GALNT14-expressing plasmids or the empty vectors (controls) and identified 305 proteins that were glycosylated to a higher degree in the presence of GalNAc-T14. Among them, 17 proteins were repetitively captured by different lectins and identified in different cell lines (Table 1). Particularly, proteins of EIF3G, SRSF8, SRP14, and ACTB were detected in five experimental settings (Table 1).

### 3.2. Tenofovir Is a Leading Drug That Down-Regulates GalNAc-T14 Substrates in a Connective Map Analysis

The connective map is an important online resource offering the relationship between diseases, genes, and drugs for biomedical research [39,40]. This system is capable of evaluating an extensive list of medicinal compounds with respect to their effects on gene expression perturbations in culture cells [39,40,41]. It is therefore ideal for finding drugs that can elicit specific alterations of a given set of genes. We therefore searched for drugs that could antagonize the GalNAc-T14 substrates in Table 1 using the connective map. A total of 1085 medicinal compounds were found to be capable of down-regulating these substrates, with different levels of strengths indicated by the weighted connectivity score in Figure 1B [41]. Among these compounds, nicardipine, atorvastatin, and tenofovir are the three leading drugs with the highest score (~100). Tenofovir is of particular interest as it is highly relevant in HBV-related HCC patients. We then scrutinized all the oral anti-HBV drugs in the connective map. Lamivudine ranked 723, while entecavir ranked 729 in their effects of suppressing expression of GalNAc-T14 substrates (Figure 1B). Adefovir and telbivudine, on the other hand, were not included in the connective map dataset. 

### 3.3. Tenofovir But Not Entecavir Hampers the Anti-Tumor Capability of Sorafenib In Vitro

**Figure 2:**
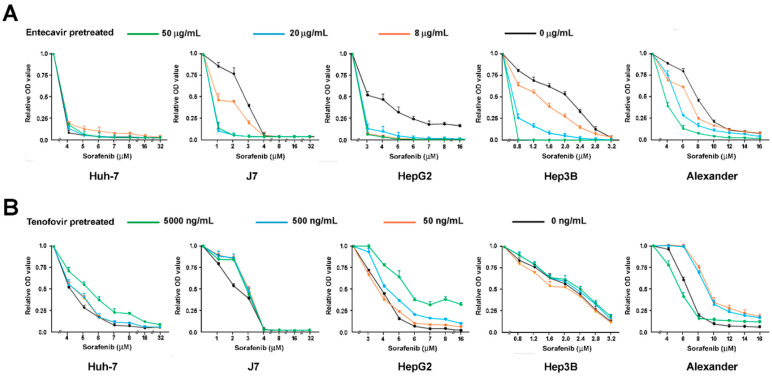
The dose-escalating assays of sorafenib treatment in reducing the viability of five different HCC cell lines, Huh7, J7, HepG2, Hep3B, and Alexander, which have been pretreated by (**A**) entecavir or (**B**) tenofovir in a wide range of concentrations.

We then investigated the viability of five different HCC cell lines (Huh7, J7, HepG2, Hep3B, and Alexander) pretreated by entecavir or tenofovir, followed by the treatment of sorafenib in different concentrations (Figure 2). We started from no pretreatment (shown as black viability curves), then increased the pretreatment doses of entecavir and tenofovir stepwise (Figure 2). The drug sensitivity to sorafenib in HCC cells increased as concentrations of entecavir increased (to a level much higher than therapeutic levels) in J7, Hep3B, and Alexander cells. In contrast, the drug sensitivity to sorafenib reduced as the concentration of tenofovir increased (1 to 2 logs of the therapeutic concentrations) in Huh7, J7, and HepG2 cells. In Alexander cells, treatment with the highest concentrations of tenofovir (green line, 5000 ng/mL) did not induce drug resistance. The in-vitro dose-escalating assays showed a tenofovir-induced resistance to sorafenib in HCC cells.

### 3.4. Patients Simultaneously Treated by Sorafenib and Tenofovir Have Shorter Overall Survival than Those Treated by Sorafenib and Entecavir

The in vitro interactive effect of tenofovir and sorafenib motivated us to investigate real-world clinical data. A total of 181 HBV-related HCC patients treated by sorafenib in Chang Gung Memorial Hospital were enrolled (Table 2). The average age at the start of sorafenib treatment was 60.4 years old. The majority of patients were male (88.4%, Table 2). Twelve patients were co-infected by hepatitis C (6.6%). No patient was co-infected by human immunodeficiency virus (HIV). A total of 67 patients were alcoholic (37.0%). Liver cirrhosis was found in most of the patients (84.0%). A total of 63 patients received no oral-antiviral drugs during the sorafenib treatment (35.4%). A total of 96 patients received entecavir (53.0%), while 22 patients received tenofovir (12.2%). No significant differences in clinical characteristics were found between these patients receiving different treatments (Table 2).

**Figure 3:**
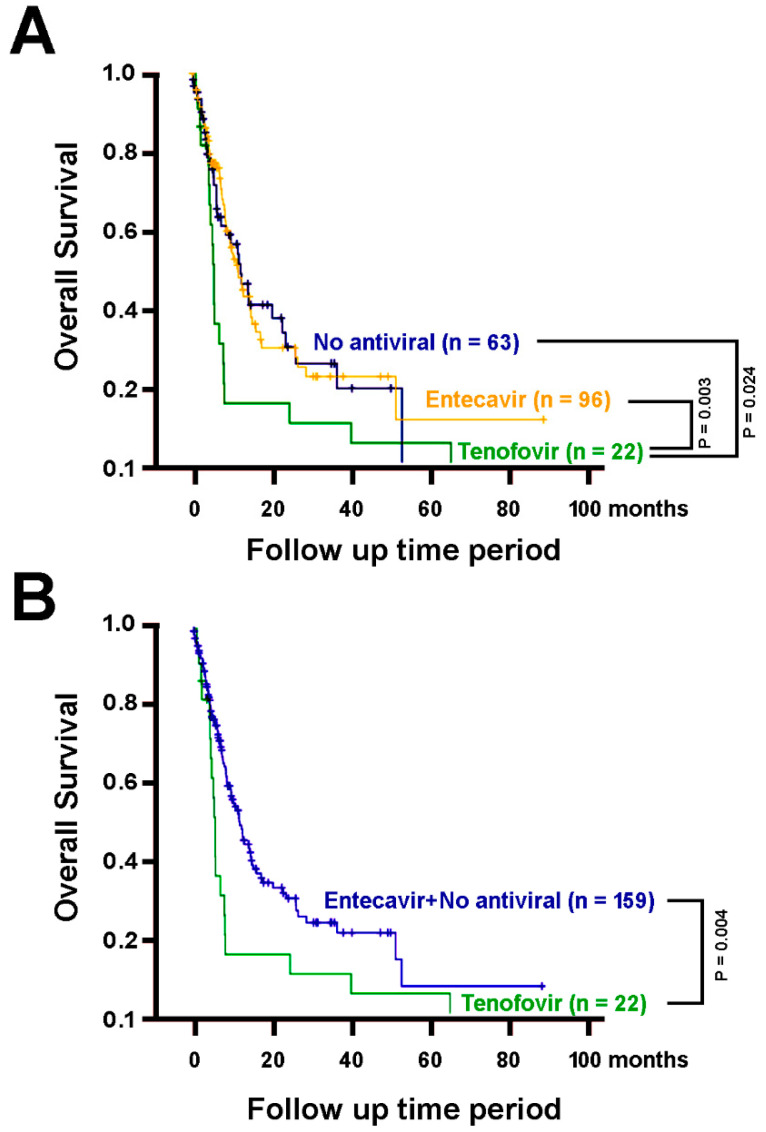
The overall survival of patients treated by sorafenib alone (*n* = 63) or in combination with entecavir (*n* = 96) or tenofovir (*n* = 22). (**A**) Patients with tenofovir treatment, in addition to sorafenib treatment, have significantly poorer overall survival than those with no oral-antiviral treatments (*p* = 0.024), with entecavir (*p* = 0.003). (**B**) Patients with tenofovir treatment have significantly poorer overall survival than the group comprising both those without oral-antiviral treatments and those with entecavir (*p* = 0.004).

We performed a Cox proportional hazards model analysis for the clinical factors of these patients with respect to the overall survival. Among them, liver cirrhosis, distant metastasis, ascites, albumin concentrations, bilirubin, AST, ALT, neutrophil, hemoglobin, and tenofovir co-treatment manifested statistically significant association with overall survival (Table 3). As such, tenofovir co-treatment was a previously unrecognized predictive factor for shorter overall survival (Hazard ratio = 2.060, 95% confidence interval = [1.256, 3.381], *p* = 0.004). The overall survival associations of these factors were re-calculated using Kaplan-Meier plots (Figure 3). Patients with tenofovir treatment had significantly shorter overall survival than those without oral-antiviral treatments (log-rank *p* = 0.024, Figure 3A), those with entecavir (*p* = 0.003, Figure 3A), and the latter two groups combined (*p* = 0.004, Figure 3B).

## 4. Discussion

It is a mandate for clinicians to offer optimal treatments to cancer patients, especially when the treatments carry profound side effects and their efficacies are limited. The present study led to the unexpected finding that anti-HBV agents (entecavir, lamivudine, and tenofovir) had different strengths in down-regulating GalNAc-T14 substrates, whereas *GALNT14* genotypes (and thus the GalNAc-T14 levels) were associated with sorafenib treatment outcomes [29]. Lamivudine is no longer a first-line anti-HBV treatment and therefore not being investigated further in this study. Entecavir and tenofovir are currently the first-line treatments for chronic hepatitis B [16,17,18,19,20]. They are both nucleus(t)ide analogs and are commonly perceived to be equally effective in viral suppressions. We conducted a series of dose-escalating assays demonstrating that HCC cells were more resistant to sorafenib in the presence of tenofovir (pretreatment) than entecavir. Additionally, clinical investigation in patients treated by sorafenib showed that the use of tenofovir was a previously overlooked risk factor for unfavorable survival. 

Nucleus(t)ide analogs for chronic hepatitis B are often prescribed in HCC patients who remained HBV viremic. The present investigation offered the first piece of evidence that antiviral treatments could interfere with anti-HCC treatments such as sorafenib. This interference likely manifested through alterations of GalNAc-T14 substrate levels. Tenofovir can also be used to treat human immunodeficiency virus (HIV) infection. A previous small retrospective study of HIV and HCC patients treated by sorafenib and highly active anti-retroviral therapy (HAART) concluded that HAART was reasonably safe and effective [42]. However, no detailed comparisons were made between different antivirals in term of survival. In light of the present study, this issue should be clarified. 

The connective map intended to offer the gateway between diseases, genes, and drugs [39,40], by quantifying genome-wide perturbation (up-regulation and/or down-regulation) of genes at the RNA levels, measured by gene expression microarray. The system is often queried by a set of genes, to see the relative positions of these genes in the total gene list ranked by the perturbation effect, i.e., a concept similar to the gene set enrichment analysis (GSEA) [43]. Here, we utilized the list of the GalNAc-T14 substrates, which were proteins, and asked the system to find medicinal compounds that could down-regulate the RNA expression profile of genes encoding the GalNAc-T14 substrates. If the RNA is down-regulated, then the corresponding proteins should also be down-regulated. As for the molecular mechanistic scenario, there are two possibilities that tenofovir could serve as an antagonist or a mimic to GalNAc-T14. Firstly, these substrates after being O-glycosylated (by GalNAc-T14) are functionally up-regulated. Addition of tenofovir could reduce the expression of these substrates and thus counteract GalNAc-T14 function. In the literature, the ribosomal proteins RPL5 and RPL11 are critical for the proper function of the tumor suppressor Tp53 [44,45], and our substrates included RPL 3, 6, 17, 27, 27A, and P1. Impaired ribosomal proteins by somatic or germline loss-of-function mutations have been shown to associate with malignancy [44]. In this view, GalNAc-T14 promotes the tumor suppressor function, while tenofovir acts against it. Alternatively, it is also possible that O-glycosylation of these substrates leads to down-regulation of their function and thus promotes cancer growth. Previous studies showed that overexpression of GalNac-T14 promotes tumor growth and metastasis in several cancers [33,34,35]. In this view, tenofovir mimics GalNac-T14 function to promote cancer growth. At this time, it is still unclear which scenario is correct.

In the present study, after the hepatoma cells were pre-treated by tenofovir and entecavir, it was found that the cell proliferation rate did not manifest a statistically significant difference between tenofovir-treated, entecavir-treated, or untreated cells (assessed by MTT assays). Therefore, the mechanism by which tenofovir reduces the cell sensitivity to sorafenib might not be due to cell proliferation. In one of our previous publications [25], it was found that patients with the “TT” genotype in the SNP rs9679162 had better clinical outcomes, as well as higher tumor/non-tumor ratios of GalNAc-T14 protein and lower tumor/non-tumor ratios of cFLIP-S, a major anti-apoptosis protein. Down-regulation of GalNAc-T14 substrates by tenofovir might perturb this regulatory axis, resulting in enhanced anti-apoptosis effects. 

## 5. Conclusions

By glycoproteomic-identification of the GalNac-T14 substrates followed by connective map analysis, we discovered that tenofovir treatment down-regulated a subset of genes highly overlapped with the genes encoding GalNac-T14 substrates. Subsequent clinical data analysis showed that HBV/HCC patients treated by sorafenib and tenofovir had a shorter overall survival compared with those treated by sorafenib and entecavir or those receiving no antiviral treatment.

## Figures and Tables

**Table 1:** List of O-glycosylated proteins identified in the evaluation of GalNAc-T14 substrates. We evaluated three cell lines, Huh7, J7, and Mahlavu, and employed two lectins, Peanut Agglutin (PNA) and Vicia Villosa Lectin (VVA), for the capturing. Glycosylated amino acids estimated by the NetOGlyc 4.0 Server are shown together with their franking sequences.

Item	Gene Symbol	Chr	UniPort ID	SwissPort ID	Franking Sequence of Glycosylated Amino Acid	Location of Glycosylation	Protein Length	Protein Name
1	EIF3G	19	EIF3G_HUMAN	O75821	220-GASRRGESMQ-231	223	320	Eukaryotic translation initiation factor 3 subunit G
2	SRSF8	11	SRSF8_HUMAN	Q9BRL6	240-RSRSRSSSMT-251	250	282	Serine/arginine-rich splicing factor 8
3	SRP14	15	SRP14_HUMAN	P37108	120-ATAPTTAATT-131	125	136	Signal recognition particle 14 kDa protein
4	ACTB	7	ACTB_HUMAN	P60709	50-DSYVGDEAQS-61	60	375	Actin, cytoplasmic 1 (Beta-actin)
5	HNRNPA1L2	13	RA1L2_HUMAN	Q32P51	180-LPKQEMASAS-191	188	320	Heterogeneous nuclear ribonucleoprotein A1-like 2
6	NAP1L1	12	NP1L1_HUMAN	P55209	280-GRGTVRTVTK-291	284	391	Nucleosome assembly protein 1-like 1
7	RPL27	17	RL27_HUMAN	P61353	50-RKVTAAMGKK-61	54	136	60S ribosomal protein L27
8	CAPZA2	7	CAZA2_HUMAN	P47755	120-RTSVETALRA-131	123	286	F-actin-capping protein subunit alpha-2
9	YBX2	17	YBOX2_HUMAN	Q9Y2T7	300-ETKPSQGPAD-311	305	364	Y-box-binding protein 2
10	RPL6	12	RL6_HUMAN	Q02878	120-VPRKLLSHGK-131	127	288	60S ribosomal protein L6
11	YWHAE	17	1433E_HUMAN	P62258	60-RIISSIEQKE-71	65	255	14-3-3 protein epsilon
12	RPL27A	11	RL27A_HUMAN	P46776	10-LRGHVSHGHG-21	16	148	60S ribosomal protein L27a
13	RPLP1	15	RLA1_HUMAN	P05386	80-APSTAAAPAE-91	83	114	60S acidic ribosomal protein P1
14	RPL3	22	RL3_HUMAN	P39023	20-RSSRHRGKVK-31	23	403	60S ribosomal protein L3
15	TUBA3E	2	TBA3E_HUMAN	Q6PEY2	360-TVVPGGDLAK-371	361	450	Tubulin alpha-3E chain
16	RPS26	12	RS26_HUMAN	P62854	90-ARKDRTPPPR-101	96	115	40S ribosomal protein S26
17	RPL17	18	RL17_HUMAN	P18621	0-MVRYSLDPEN-11	5	184	60S ribosomal protein L17

**Table 2:** Baseline clinical characteristics of advanced HBV-related HCC patients treated by sorafenib with or without NUC.

Variables	All Patients	Entecavir Used	Tenofovir Used	No Antiviral Used	*p* *
Number of patients	181	96	22	63	
Age, years, mean ± SD	60.4 ± 12.3	58.7 ± 11.3	60.6 ± 12.9	63.0 ± 13.2	0.104
Sex, Male (%)	160 (88.4%)	87 (90.6%)	20 (90.9%)	53 (84.1%)	0.423
Anti-HCV, positive (%)	12 (6.6%)	5 (5.2%)	1 (4.5%)	6 (9.5%)	0.517
Alcoholism, Yes (%)	67 (37.0%)	37 (38.5%)	10 (45.5%)	20 (31.7%)	0.468
ECOG status, “>0” (%)	43 (23.8%)	24 (25.0%)	6 (27.3%)	13 (20.6%)	0.752
Cirrhosis, Yes (%)	152 (84.0%)	85 (88.5%)	19 (86.4%)	48 (76.2%)	0.11
Portal vein thrombosis, Yes (%)	98 (54.1%)	56 (58.3%)	14 (63.6%)	28 (44.4%)	0.145
Distant metastasis, Yes (%)	73 (40.3%)	37 (38.5%)	9 (40.9%)	27 (42.9%)	0.862
BCLC stage B, Yes (%) **	26 (14.4%)	15(15.6%)	3 (13.6%)	8 (12.7%)	0.871
Size, cm, mean ± SD	5.9 ± 4.3	5.6 ± 4.1	5.7 ± 4.6	6.5 ± 4.6	0.411
Ascites, Yes (%)	45 (24.9%)	27 (28.1%)	7 (31.8%)	11 (17.5%)	0.227
AFP, ng/mL, median (range)	241.8 (0.8 to 831318)	238.4 (1.7 to 831318)	216.9 (4.7 to 84144)	259.0 (0.8 to 510606)	0.277
HBV-DNA, × 10^6^ copies/mL, median (range) #	0.0 (0.0 to 112.9)	0.0 (0.0 to 0.0)	0.0 (0.0 to 0.0)	0.0 (0.0 to 112.9)	0.145
Albumin, g/dL, mean ± SD	3.7 ± 0.6	3.8 ± 0.5	3.8 ± 0.6	3.7 ± 0.7	0.32
Bilirubin, mg/dL, mean ± SD	1.2 ± 1.7	1.1 ± 2.0	1.2 ± 0.6	1.2 ± 1.4	0.934
Prothrombin time, sec, mean ± SD	12.2 ± 1.7	12.4 ± 2.0	11.9 ± 1.2	11.9 ± 1.2	0.143
Creatinine, mg/dL, mean ± SD	0.9 ± 0.6	0.9 ± 0.7	0.9 ± 0.4	0.9 ± 0.4	0.995
AST, U/L, mean ± SD	74.6 ± 68.4	75.9 ± 76.2	72.9 ± 44.2	73.2 ± 63.2	0.966
ALT, U/L, mean ± SD	46.0 ± 38.3	45.5 ± 42.0	53.2 ± 44.0	44.3 ± 29.5	0.634
White blood cell, 10^9^/L, mean ± SD	6.3 ± 3.0	6.1 ± 2.9	5.4 ± 2.0	6.9 ± 3.3	0.108
Neutrophil, percentage, mean ± SD	66.1 ± 12.9	66.1 ± 13.8	62.2 ± 11.7	67.3 ± 11.7	0.271
Hemoglobin, g/dL, mean ± SD	12.4 ± 2.0	12.4 ± 2.1	12.4 ± 1.5	12.3 ± 2.0	0.999
Platelet, 10^9^/L, mean ± SD	168.3 ± 93.2	162.3 ± 90.2	143.5 ± 89.8	186.2 ± 97.1	0.118
Previous treatment, Yes (%)	161 (89.0%)	89 (92.7%)	20 (90.9%)	52 (82.5%)	0.129

* *p*: the significance level for comparing the entecavir used, tenofovir used, and no antiviral used patients. Chi-square tests were used for categorical data, while analysis of variance (ANOVA) tests were used for numerical data. HCV, hepatitis C virus; ECOG, Eastern Cooperative Oncology Group; AFP, alpha-fetoprotein; AST, aspartate transaminase; ALT, alanine transaminase. **: The remaining patients were all BCLC stage C. #: The lower quantification limit was 116 copies/mL. All patients treated by entecavir or tenofovir had HBV-DNA < lower quantification limit.

**Table 3:** Cox proportional hazard analysis in 181 HBV-related HCC patients for clinical variables in relationship to overall survival.

Variables	HR (95% CI)	*p*
Age, per year	0.992 (0.997, 1.008)	0.344
Sex, Male = 1	0.741 (0.405, 1.356)	0.331
Anti-HCV, positive = 1	0.736 (0.300, 1.809)	0.505
Alcoholism, positive = 1	1.233 (0.842, 1.806)	0.281
ECOG status, “>0” = 1	1.324 (0.845, 2.075)	0.221
Cirrhosis, Yes = 1	2.160 (1.182, 3.946)	**0.012**
Portal vein thrombosis, Yes = 1	1.098 (0.755, 1.599)	0.624
Distant metastasis, Yes = 1	1.488 (1.023, 2.164)	**0.038**
Size, per cm	1.020 (0.978, 1.065)	0.353
Ascites, Yes = 1	2.535 (1.676, 3.833)	**<0.001**
AFP, per 1000 ng/mL	1.001 (0.999, 1.003)	0.234
Albumin, per g/dL	0.532 (0.380, 0.745)	**<0.001**
Bilirubin, per mg/dL	1.340 (1.175, 1.528)	**<0.001**
Prothrombin time, per sec	0.995 (0.877, 1.129)	0.941
Creatinine, per mg/dL	1.176 (0.839, 1.650)	0.347
AST, per U/L	1.008 (1.005, 1.010)	**<0.001**
ALT, per U/L	1.006 (1.002, 1.011)	**<0.006**
White blood cell, per × 10^9^/L	1.069 (0.999, 1.144)	0.053
Neutrophil, per percentage	1.032 (1.015, 1.050)	**<0.001**
Hemoglobin, per g/dL	0.863 (0.782, 0.953)	**<0.003**
Platelet, per × 10^9^/L	0.999 (0.997, 1.002)	0.611
Previous treatment, Yes = 1	1.052 (0.596, 1.855)	0.862
Tenofovir used, Yes = 1	2.060 (1.256, 3.381)	**<0.004**
Entecavir used, Yes = 1	0.798 (0.549, 1.159)	0.235
No antiviral used, Yes = 1	0.797 (0.533, 1.191)	0.268

HCV, hepatitis C virus; ECOG, Eastern Cooperative Oncology Group; AFP, alpha-fetoprotein; AST, aspartate transaminase; ALT, alanine transaminase. *p* values below 0.05 are shown in bold face.

## Data Availability

Data will be available for academic scientists upon reasonable request to the corresponding author.
